# The Effectiveness of Teacher Support for Students’ Learning of Artificial Intelligence Popular Science Activities

**DOI:** 10.3389/fpsyg.2022.868623

**Published:** 2022-06-03

**Authors:** Sheng-Yi Wu, Kuay-Keng Yang

**Affiliations:** ^1^International Master Program in STEM Education, National Pingtung University, Pingtung City, Taiwan; ^2^Department of Science Communication, National Pingtung University, Pingtung City, Taiwan

**Keywords:** artificial intelligence, popular science activities, teacher support, informal curriculum, STEM education, universal education

## Abstract

The burgeoning of new technologies is increasingly affecting people’s lives. One new technology that is heatedly discussed is artificial intelligence (AI) in education. To allow students to understand the impact of emerging technologies on people’s future lives from a young age, some popular science activities are being progressively introduced into elementary school curricula. Popular science activities are informal education programs and practices of universal education. However, two issues need to be discussed in the implementation of these activities. First, because these informal curricula are usually short in duration, the question of whether they only serve to generate motivation or actually enhance learning outcomes requires examination. Second, the role of teacher support in popular science activities and its impact on students’ learning results need to be further investigated. To this end, this study aims to explore the effectiveness of popular AI science activities in informal curricula on students’ AI achievement and the interrelationship between students’ learning outcomes in popular AI science activities with and without teacher support. A 6-h-long AI popular science activity was conducted with 22 fifth- and sixth-grade students in elementary school. This study was conducted using a one-group pretest and posttest design, and the data collection tools included AI achievement pre- and posttests and an artifact scoring rubric. The results showed that with regard to learning outcomes, popular science activities were helpful for cognitive enhancement of AI concepts, but more time was needed for skills to improve. Additionally, this study found that students’ learning performance was different with and without teacher support. Activities with teacher support can enhance students’ learning outcomes, but students become accustomed to relying on their teachers. In contrast, activities without teacher support seem to be more effective in fostering students’ independent computational thinking and problem-solving abilities.

## Introduction

### Emerging Technological Development and Artificial Intelligence Education

Due to the continuous advancement of technologies and techniques, people are increasingly interested in emerging technologies. This term refers to new technologies that are characterized by radical novelty, relatively fast growth, coherence, prominent impact, uncertainty, and ambiguity (Rotolo et al., 2015). Currently, several emerging technologies affect people’s lives, such as artificial intelligence (AI; Haenlein and Kaplan, 2019), 5G/6G communication networks (Sheth et al., 2020), and quantum computers (Alvarez-Rodriguez et al., 2018). To provide students with an early understanding of the impact of these technologies on people’s future lives, popular science activities are slowly being introduced into the learning activities of elementary schools. Currently, one of the most commonly discussed issues is AI education (Kim and Park, 2017; Lin et al., 2021).

Among many issues involved in emerging technologies, the topic of AI has had a strong impact on life in recent years. The term “AI” first appeared at a 1956 Dartmouth conference (Bell, 2021). There are many definitions of AI, but the term is generally known to the public as a technology that simulates human-like intelligence through computer programs (Russell and Norvig, 2016). In addition to active research in AI and the expansion of relevant industries in recent years, many scholars have been exploring the application of AI in education (AIED). For example, Baker (2000) suggested that in the near future, AI applications in education will include models as scientific tools, models as components of educational artifacts, and models as bases for the design of educational artifacts. Colchester et al. (2017) also analyzed the current status of the use of learning systems through AI and noted that AI can assist in adapting learning styles. After analyzing learners’ learning models, AI can design appropriate methods of teaching and allow learners to study by themselves.

For elementary school students, the use of AI seems difficult. However, this perception has slowly changed. The content of AI education emphasizes AI popular science education rather than AI technology (Geraci, 2011; Zhang et al., 2021). Since AI is an important issue that affects current and future life, the goal of teaching it is not for students to take exams but to nurture students’ awareness of the influence of AI on life and improve their logical thinking and problem-solving (Lin et al., 2021). Therefore, the promotion of AI education from a young age is a matter of great urgency. To date, however, AI-supported emerging technologies cannot be integrated into the formal curriculum and can only be taught in informal curricula (i.e., popular science activities).

### Improvement of Scientific Literacy Through Popular Science Activities

As human life continues to evolve and prosper at a rapid pace, scientific literacy has become a necessary capacity for society at large. According to the National Science Teachers Association (1982), a scientifically literate citizen must (1) understand the interaction between science, technology, and society and actively inquire and solve related problems; (2) have the ability to comprehend and apply scientific concepts, theories, and facts; (3) understand the essential spirit of science and make positive and meaningful judgments about social issues; (4) apply scientific methods, concepts and correct scientific attitudes to solve everyday problems; and (5) have a deeper understanding and more active involvement in the relationship between science, technology, and the environment. From the above description of scientific literacy, it is apparent not only that scientific literacy is required for professionals but also that every citizen needs information on this knowledge and related concepts and attitudes.

The goal of scientific development has gradually shifted from elite education to the cultivation of scientific literacy for all. Therefore, popular science activities act as an important channel to promote education for higher scientific literacy among all people. According to Dzan et al. (2015), popular science activities can increase students’ willingness to learn science by allowing them to engage in fun and interesting activities and broadening their knowledge.

Since AI affects our lives, it is important for AI education to enhance students’ awareness and knowledge of AI through interesting activities from a young age to promote universal scientific literacy (Hsu et al., 2022). However, because popular science activities are not part of the formal curriculum, they are often conducted as experiential events and as part of non-standard courses. Generally, experiential activities are scheduled for approximately 20–60 min, while informal curricula may be scheduled for several hours.

### Effectiveness of Popular Science Activities in Informal Curricula

Informal curricula refer to educational activities that take place in off-campus settings (Jeffs and Smith, 2021). Informal curricula can be implemented in a variety of ways, including a series of lessons, seminars, short courses, and experiential activities. Popular science activities are often conducted in these common formats to disseminate scientific knowledge. In addition to other types of activities, popular science activities are often designed using theories, such as hands-on learning (Dewey, 1997), experiential learning (Kolb, 1981), or STEM learning frameworks (Kelley and Knowles, 2016).

The purpose of designing curricula based on relevant theories is to elicit motivation and learning outcomes in science subjects *via* popular science activities. Nugent et al. (2010) and Anand and Dogan (2021) note that the purpose of learning activities in informal curricula is to provide students with opportunities to engage more deeply in learning activities. Lin et al. (2015) arranged popular science activities through the Nanotechnology-based Popular Science Education Promotion and Teaching (NPSEPT) program. The results showed that the participants, elementary school students, achieved significant gains in nanotechnology learning performance and outcomes. The effectiveness of these popular science activities was attributed to the curriculum design and students’ willingness to learn. The National Research Council (2000) emphasized in the “schema for learning sciences” that when students identify themselves as science learners in an informal curriculum, this facilitates their learning of science.

Although some of the above studies have confirmed the effectiveness of popular science activities for enhancing science learning, some controversies remain. Currently, some popular science activities are promoted in the form of short courses. Because of time constraints, instructors are unable to fully impart science knowledge and guide inquiry and hands-on activities (Hight et al., 2021; Shu et al., 2021). Therefore, there is a paucity of information on whether popular science activities of short duration (a few hours) can help students achieve learning outcomes and motivate them for science studies.

On the other hand, previous studies have shown that popular science activities are effective in increasing students’ interest in and motivation for learning (Nugent et al., 2010; Lin et al., 2015; Anand and Dogan, 2021). However, popular AI science activities require high cognitive abilities, and students need to spend time writing programs (i.e., using computational thinking skills) in addition to assembling simple building block models (e.g., assembling machines). This study also investigated the effect of short hands-on AI activities on students’ interest in learning. Therefore, the first question of this study concerns the impact of short-term popular science activities in informal curricula on students’ AI achievement tests.

### The Impact of Teacher Support

Teacher support includes both academic and emotional support (Patrick et al., 2007; Liu et al., 2018). Teacher academic support refers to students’ perception that teachers care about what and how much they learn, while teacher emotional support reflects students’ awareness that teachers care about students as individuals (Johnson et al., 1985). Many studies have confirmed the positive impact of teacher support on student learning. For example, Yu and Singh (2018) indicated that teacher support indirectly affects students’ mathematics achievement through their sense of mathematics self-efficacy and affects students’ interest in mathematics courses. Fredricks et al. (2018) examined how motivation and background influence boys’ and girls’ engagement in mathematics and science subjects. The results indicated that motivation and background factors were significantly associated with students’ engagement level, with girls being more likely to participate in subject activities because of teacher support. Li et al. (2021) also noted that teacher support of literacy development can improve students’ ability to build scientific models.

From the above literature, it is clear that teacher support is helpful to students (Marec et al., 2021). In the case of popular science activities, it is worthwhile to explore the ways in which teacher support is incorporated into students’ research practices. During such activities, because of the limited time, students often opt for teacher support when they encounter problems. However, in the case of AI hands-on activities, does the teacher’s guidance of students in programming limit students’ ability to try to develop computational thinking on their own? Is it the right choice to give direct answers to students when learning about AI? According to the above literature, the benefits of teacher support mostly appear in terms of motivation, self-efficacy, and interest in learning; in regard to cognitive learning outcomes, teacher support serves only to improve student learning overall. However, there is insufficient research on the impact of teacher support on students’ thinking patterns (e.g., creativity and logical thinking). Current popular science activities, as informal curricula, are intended not only to enhance students’ interest in learning science but also to help their thinking patterns evolve. Teacher support is expected to help students learn more effectively (Sadoughi and Hejazi, 2021), but there is a possibility that students may not be able to think and investigate independently in certain areas because of their reliance on teachers (e.g., Ma et al., 2021). Therefore, the second question pursued in this study concerns the relationship between the presence or absence of teacher support and students’ learning outcomes.

### Research Questions

In summary, there are two main issues involved in conducting AI-related popular science activities in informal curricula. The first issue is whether short popular science activities only spur motivation and interest in learning or whether they also provide additional enhancement to learning outcomes. The second issue is that while teacher support in science education has been recognized by many scholars as contributing to students’ learning results, the role of teacher support in popular science activities needs to be further explored. The following are the research questions for this study:

1. What is the effectiveness of popular AI science activities in informal curricula on students’ AI achievement (i.e., AI achievement pre- and posttests)?
2. What is the interrelationship among students’ learning outcomes (i.e., artifact scoring rubric) in popular AI science activities (i.e., creativity, computational thinking, problem-solving, and the completeness of finished work) with and without teacher support?

## Research Design

To investigate the above two questions, this study conducted a 6-h-long popular science activity in AI education, including AI knowledge, coding, and AI visual recognition chip applications and problem-solving through programming. This study was conducted using a one-group pretest and posttest design, and the design is described below.

### Participants and Grouping

The participants of this study were elementary school students in grades 5–6 who applied to participate voluntarily. All of the participants had basic knowledge about a visual programming language, for example, Scratch. A total of 22 students, including 16 boys and six girls, participated in the program; they were divided into 11 random pairs during the activity.

### Research Procedures

**Figure 1:**
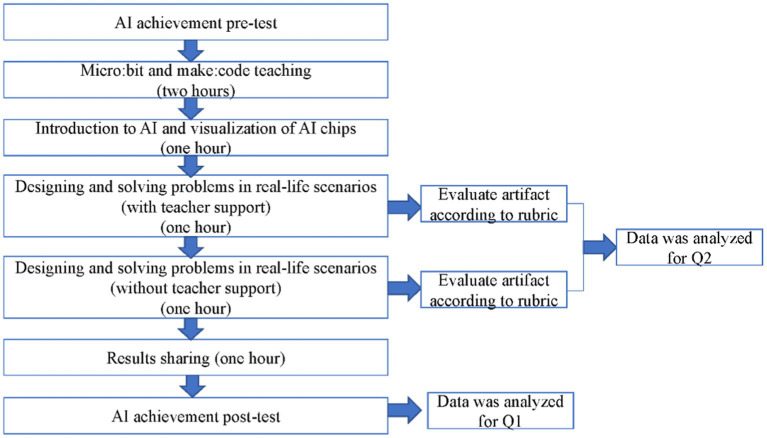
Experimental flow chart.

To investigate the two research questions, a 6-h AI education activity was conducted in 1 day. This activity was designed based on the STEM learning conceptual framework (Kelley and Knowles, 2016) and project-based learning (Triana et al., 2020) with both teaching sessions and hands-on activities. AI achievement pre- and posttests were arranged. In addition to lectures and hands-on exercises, group problem-solving activities were arranged (Figure 1). The schedule of activities included (a) Micro:bit and makecode teaching, (b) introduction to AI and the visualization of AI chips, (c) designing and solving problems in real-life scenarios (with teacher support), (d) designing and solving problems in real-life scenarios (without teacher support), and (e) result sharing.

**Figure 2:**
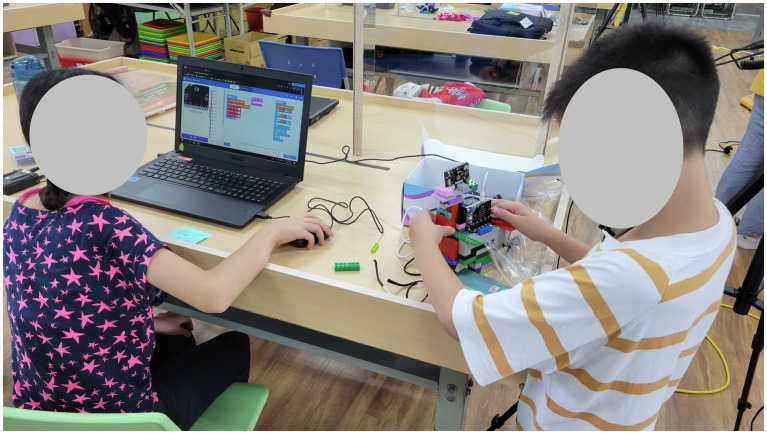
Real-life problem design and solution activities.

In the activities on problem design and problem-solving in real-life scenarios, one activity was conducted with teacher support. In this activity, students could ask questions and the teacher provided prompts; if students did not understand or could not perform the task, the teacher provided answers. The second activity was conducted without teacher support, meaning that students were not allowed to ask any questions to the teacher or other groups during the activity (Figure 2).

Regarding the practices of designing and solving problems in real-life scenarios, the questions included face recognition (without teacher support) and color recognition (with teacher support), and the contexts were established as follows.

- Without teacher support (face recognition): This year, the COVID-19 pandemic hit hard, and everyone has to wear a face mask when going out. However, people can often forget to do so when they are in a hurry. Therefore, a face mask reminder machine can be handy for alerting people if they are not wearing a mask when leaving home by letting out a warning sound and displaying an X on the screen. If they are wearing a mask, then the machine displays ✓ and lets out no sound. Use blocks to make a visual AI chip holder that can be loaded with the chip and place it on the desktop at just the right height to allow the visual AI chip to sense whether the student is wearing a mask when sitting.
- With teacher support (color recognition): Xiao Ming is going to a store today to buy something. The store uses color labels as price markers, including red labels for NTD 10, green labels for NTD 20, and blue labels for NTD 30. However, every time Xiao Ming buys something, he forgets the price indicated by each color. Let us use the AI visualization chip with Micro:bit to help Ming record the color price so he can easily calculate how much the items in his shopping cart cost. Use blocks to make 3–6 products and put price tags in front of the products. Red labels indicate NTD 10, green labels indicate NTD 20, and blue labels indicate NTD 30. Next, use the chip to study 1 red, 2 green, and 3 blue in order with the Micro:bit program. Press Button A to return to zero and restart the calculation. When the AI chip detects the color label, the Micro:bit displays the price of that color. If you want to buy the item, press the B button to confirm the purchase, and then add the numbers for the current total amount.

### Instrument

#### AI Teaching Aids

**Figure 3:**
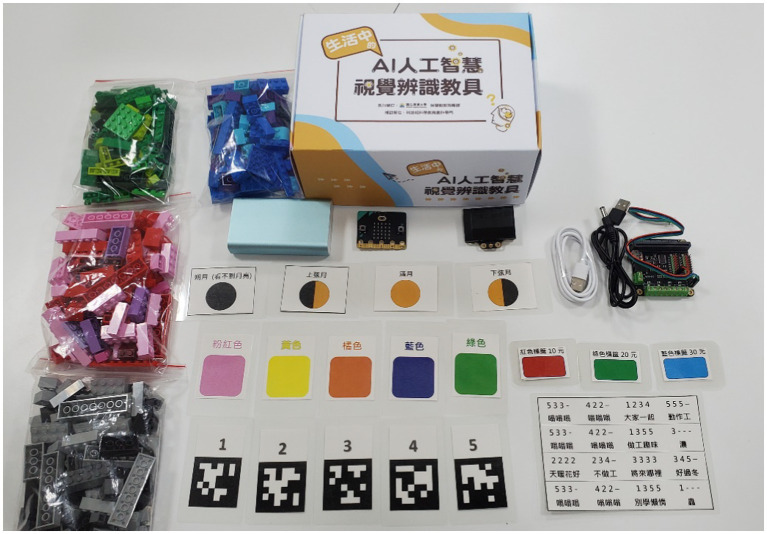
AI teaching aid kit.

The AI teaching aids developed and designed by our research team emphasize learning functions and problem-solving in real-life situations, featuring real applications, self-designed teaching instruments, and chips that can present study functions. The contents of the teaching aids (e.g., Figure 3) include Micro:bit, HuskyLens PRO AI chip, Micro:bit extensions, cables, building blocks, mobile power, and activity labels.

#### AI Achievement Pre- and Posttests

To understand the effectiveness of popular AI science activities on students’ learning of AI-related knowledge and skills, this study developed AI pre- and posttest questions. The AI pre- and posttests consisted of 10 questions on three dimensions: machine learning history, AI concepts, and coding. Three to four questions were used for each dimension.

In this study, the AI pretest and posttest questions were designed based on the AI education popular science activities textbook. After the design was completed, two elementary school teachers with experience in related fields were invited to revise the questions based on difficulty and suitability. After consensus was reached, questions from each dimension were randomly assigned to the pre- or posttest. Table 1 presents one of the questions for each dimension.

**Table 1:** Examples of AI pre- and posttest questions.

Dimension	Sample questions
Machine learning history	Which of the following is not a major area of input for the second AI development wave? deep learning expert system speech recognition artificial neural network
AI concept	Which of the following is not a main process of face recognition? face detection feature extraction feature fusion face recognition
Coding	What will be displayed when the code of the image is executed? X 50 60 Pass 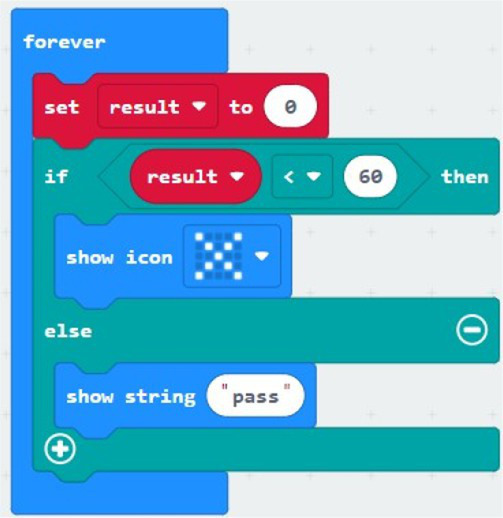

#### Artifact Scoring Rubric

In the AI popular science activity, students need to have coding skills, problem-solving skills, and creativity in addition to using AI lenses, Micro:bit, building blocks, and other related teaching aids to complete their tasks. To rate students’ ability to solve the AI artifact problems in real-life scenarios, this study referred to several studies on manual work and computational thinking (e.g., Tang et al., 2020; Li et al., 2021) and the computational thinking scale proposed by Korkmaz et al. (2017). Korkmaz et al. categorized computational thinking into five categories (i.e., creativity, algorithmic thinking, problem-solving, critical thinking, and cooperation). This study intended to evaluate students’ computational thinking *via* their AI artifact. The content of the work included the use of engineering design blocks, chip applications, and coding. Thus, in this study, two elementary school teachers and one AI expert developed and revised a real-life scenario problem-solving AI artifact scoring rubric based on the studies of Korkmaz et al. Since the artifact of this study was static, this study did not record the group as it performed the task. Therefore, it could not measure students’ critical thinking ability and cooperation ability, and these two dimensions were removed. In addition, students’ artifacts were completed to different degrees, so a category for degree of completion was added. In the scoring section, the work was rated on a scale of 0–10, with reference scoring points of 0, 5, and 10 points. Regarding reliability and validity, this study conducted content validity through relevant literature, expert validity was conducted by two primary school teachers and two university teachers (i.e., the authors), and reliability analysis was conducted through rater consistency. The final version of the rubric is shown in Table 2.

**Table 2:** Artifact scoring rubric.

Vector	Content	Rating
Creativity	Engineering design and programming enrichment and creativity	0 Uncompleted block assemblage/programs 5 Half assembled/written; incomplete structure/programs 10 Blocks assembled with creativity and complete program structure in line with the given scenario
Algorithmic thinking	Program logic and coding	0 Illogical or inapplicable program 5 There is a glitch in the logic or the program does not run 10 Complete program that can function smoothly
Problem-solving skills	Problem-solving with proper programs, blocks, and chips	0 The overall solution cannot solve the problem effectively 5 The problem is only half solved, with some loopholes 10 The overall solution can solve the problem effectively
Degree of completion	Overall integrity of service	0 The final representation is not related to the topic 5 The work is more than half wrong or lacks cohesion 10 Complete and coherent work in line with the topic that can solve the problem

### Data Analysis

For the two research questions, the data analysis was divided into two parts. The first part focused on exploring the effectiveness of students’ participation in popular AI science activities. Thus, all the participants were asked to respond to the AI achievement pre- and posttest, and then the dependent *t*-test was conducted to analyze students’ performance growth.

The second part focused on comparing the activities with and without teacher support and examining the interrelationship among students’ learning outcomes in AI popular science activities (i.e., creativity, computational thinking, problem-solving, and the completeness of finished work). Thus, the AI artifacts presented by the group (including face recognition and color recognition) were analyzed with the developed scoring rubric. Two teachers in a related field in elementary school evaluated the work according to the rubric. The interrater reliability was 0.80. The average of the two teachers’ evaluations was used as the final grade.

## Results and Discussion

This section presents and discusses the results of the data analysis based on the two research questions through the lenses of learning achievement and teacher support.

### Learning Achievement

The dependent *t*-test was conducted to understand student AI performance growth after joining the AI activities (Table 3). There is a significant difference in the overall learning effectiveness in terms of knowledge and skills in AI (*t* = −3.55, *p* = 0.002). Furthermore, a closer look at individual items reveals major differences among the dimensions: machine learning history (*t* = −3.81, *p* = 0.001) and AI concepts (*t* = −2.61, *p* = 0.016) both demonstrate improvement, while there are no significant gains for coding (*t* = 1.28, *p* = 0.213).

**Table 3:** Results of the dependent *t*-test of students pre- and post-AI achievement test.

	Machine learning history	AI concepts	Coding	Total
	Mean/Average	Mean/Average	Mean/Average	Mean/Average
Pretest	0.05/0.21	0.23/0.43	0.45/0.51	3.23/0.40
Posttest	0.45/0.51	0.64/0.49	0.27/0.46	5.05/0.44
*t* value	−3.81	−2.61	1.28	−3.55
Significance	0.001	0.016^*^	0.213	0.002

* *p* < 0.5.

It can be concluded that students’ learning of emerging AI technologies can be enhanced through 6 h of popular AI science activities combined with lectures and hands-on activities in groups. However, coding does not show such major improvement, probably because coding requires longer periods of practice and development and the short hands-on activities cannot improve students’ learning effectiveness in coding skills.

### Teacher Support

With regard to teacher support, the literature review showed that students’ learning outcomes are better in learning activities with teacher support (Fredricks et al., 2018; Yu and Singh, 2018; Li et al., 2021). Table 4 shows that the scores of activities with guidance from the teacher are higher than those with no teacher support. Next, the role of teacher support in learning the emerging technologies of AI was explored.

**Table 4:** Teachers’ activity evaluation sheet for each group.

	G1	G2	G3	G4	G5	G6	G7	G8	G9	G10	G11	Average
*Activities are supported by teachers*												
Creativity	7.5	10	8	9.5	9	6	9.5	8.5	7.5	9	6.5	8.27
Algorithmic thinking	10	9.5	9	10	9	9.5	9.5	9.5	9.5	9.5	9.5	9.50
Problem-solving skills	9	9.5	9	10	8.5	7.5	9.5	9	9	8.5	9.5	9.00
Degree of completion	9	9.5	9	10	8.5	7.5	9.5	9	7.5	7.5	9	8.73
Total	35.5	38.5	35	39.5	35	30.5	38	36	33.5	34.5	34.5	35.50
*Activities are NOT supported by teachers*												
Creativity	6	9.5	5.5	8	8	7.5	9	6	3.5	3.5	3.5	6.36
Algorithmic thinking	7.5	10	8.5	8	10	8	10	6	0.5	4.5	3	6.91
Problem-solving skills	8	10	8.5	8.5	8.5	7.5	9.5	6.5	0.5	3	3	6.68
Degree of completion	7	9	7	8.5	8.5	7.5	10	5.5	3	3	3	6.55
Total	28.5	38.5	29.5	33	35	30.5	38.5	24	7.5	14	12.5	26.50

The comparison of activities with and without teacher support shows that when students are provided with teacher assistance, work completion (*r* = 0.27), problem-solving ability (*r* = 0.35), and creativity (*r* = 0.00) are not significantly correlated with the algorithmic thinking skills (Table 5), whereas when students are not provided with teacher support (Table 6) and need to work alone in small groups, completion (*r* = 0.93), problem-solving (*r* = 0.97), and creativity (*r* = 0.87) are all highly correlated with algorithmic thinking skills.

**Table 5:** Activities supported by teachers.

	Creativity	Algorithmic thinking	Problem-solving skill	Degree completion	Average
Creativity	1				
Algorithmic thinking	0.00	1			
Problem-solving skills	0.52^*^	0.35	1		
Degree of completion	0.51^*^	0.27	0.81^**^	1	
Total	0.83^**^	0.32	0.86^**^	0.86^**^	1

* *p* < 0.5;

** *p* < 0.1.

**Table 6:** Activities NOT supported by teachers.

	Creativity	Algorithmic thinking	Problem-solving skill	Degree completion	Average
Creativity	1				
Algorithmic thinking	0.87^**^	1			
Problem-solving skill	0.89^**^	0.97^**^	1		
Degree of completion	0.96^**^	0.93^**^	0.95^**^	1	
Average	0.95^**^	0.98^**^	0.98^**^	0.98^**^	1

** *p* < 0.1.

The above analysis indicates that teacher support is related to better learning outcomes in AI popular science activities. However, an interesting finding is that students’ algorithmic thinking performance is not significantly correlated with creativity, problem-solving ability, or work completion in the presence of teacher support. The researchers theorize that when students encounter problems and teacher support is available, they become accustomed to seeking help from teachers rather than leveraging their individual algorithmic thinking ability.

On the other hand, when teacher support is not available, students’ algorithmic thinking performance is highly correlated with their creativity, problem-solving ability, and work completion on AI projects. This means that without teacher support, students must rely on their individual algorithmic thinking to solve problems. Furthermore, students’ creative performance and work completion are highly correlated with algorithmic thinking.

From these two studies, it is clear that hours long AI popular science activities are beneficial to students’ cognitive and skill acquisition. However, excessive support from teachers in the curriculum can limit students’ opportunities to practice their own algorithmic thinking. In other words, the design of future curricula needs to consider the level of teacher support and understand its impact on refining students’ high-level algorithmic thinking. Therefore, although popular science activities are conducted in a relatively short period of time, they can enhance students’ learning outcomes if they are designed appropriately. Second, learning activities with teacher support can enhance students’ learning outcomes, but they allow students to become accustomed to relying on their teachers and not think independently about the activities. In contrast, popular science activities without teacher support seem to be more effective in fostering students’ independent computational thinking and problem-solving abilities because they need to be completed independently.

## Conclusion and Suggestions

This study was conducted with the aim of understanding the impact of AI popular science activities as informal curricula on students’ learning outcomes and the relationship between the presence or absence of teacher support and learning effectiveness. This study was conducted through a 6-h-long AI popular science activity program with the participation of a total of 22 elementary school students.

The results of this study show that regarding the first research question, AI popular science activities are helpful in terms of cognitive enhancement, while more time may be needed for skill enhancement. In other words, although popular science activities are conducted in a short period of time, they can enhance students’ learning results with an appropriate curriculum design. For the second research question, AI science education activities with and without teacher support have different learning outcomes. AI science education activities with teacher support lead to higher scores on students’ work, that is, the quality of work is better. In the case of AI science education activities without teacher support, although the quality of students’ work is not as good, students can rely on their own computational thinking skills to complete the tasks and perform better in creativity and problem-solving skills, which is another indicator of learning effectiveness. Teacher support is similar to teachers building scaffolds in students’ zone of proximal development (ZPD; Xi and Lantolf, 2021), but it is worth studying how teachers can build a scaffold to avoid making students overly dependent on teachers for learning. Therefore, future research could investigate the effects of different levels of teacher support on students’ AI knowledge and higher-level cognitive abilities to provide a reference for curriculum design.

Based on the above research findings, in the era of universal education, the results of this study suggest that popular science activities are indeed helpful for academic learning achievement. In addition, results and suggestions for the on-site evaluation of teacher support in popular science activities are presented. Based on the above results and discussion, this study offers several suggestions for practice. First, in informal curricula, interesting popular science activities about emerging technologies can increase students’ motivation to learn (Dzan et al., 2015). In terms of learning outcomes, there is a close relationship between activity time and curriculum design. It is unclear how much time is required for teacher-led, hands-on, and collaborative learning experiences. However, it is confirmed that curriculum design needs to take these processes into account. In addition, based on retrieval practice effect theory (Weinstein et al., 2018), more practice time may be required when aiming for skill-based learning outcomes. Second, since AI science education activities with and without teacher support have different implications for learning, it is recommended to include both activity designs in the curriculum. In general, it is best to arrange science education activities with teacher support in the early stages to provide students with teacher guidance first and help them achieve some initial success. Then, students can be given more learning tasks without teacher support to enhance their independent thinking and problem-solving skills through competitions or awards and to elicit their computational thinking capacities, creativity, and problem-solving skills.

Finally, this study was a pilot study. In terms of research limitations, because only 22 students were included and the instrument used to measure effectiveness was developed by the research team, the results of this study should be compared with those of similar studies in the future. In addition, it is recommended that future studies (a) use a larger sample pool and longer study time, (b) use standardized scales or rubrics for distinct popular science activities, and (c) adopt attitudinal questionnaires, behavioral observations (e.g., lag sequential analysis, Wu, 2020), or physiological sequences (Wu and Su, 2021) in addition to tests and work evaluations to measure learning effectiveness.

## Data Availability Statement

The raw data supporting the conclusions of this article will be made available by the authors, without undue reservation.

## Ethics Statement

The studies involving human participants were reviewed and approved by National Cheng Kung University Governance Framework for Human Research Ethics. Written informed consent to participate in this study was provided by the participants’ legal guardian/next of kin.

## Author Contributions

S-YW proposed the study design, conducted the experiments, analyzed the data, and wrote the manuscript. K-KY assisted in data analysis and manuscript writing. All authors contributed to the article and approved the submitted version.

## Funding

This research is partially supported by the Ministry of Science and Technology (MOST) and National Pingtung University, Taiwan, R.O.C. under grant MOST 109-2511-H-153-005- MY2 and NPTU-110-007.

## Conflict of Interest

The authors declare that the research was conducted in the absence of any commercial or financial relationships that could be construed as a potential conflict of interest.

## Publisher’s Note

All claims expressed in this article are solely those of the authors and do not necessarily represent those of their affiliated organizations, or those of the publisher, the editors and the reviewers. Any product that may be evaluated in this article, or claim that may be made by its manufacturer, is not guaranteed or endorsed by the publisher.
